# Management of uncomplicated malaria in private health facilities in North-West Ethiopia: a clinical audit of current practices

**DOI:** 10.1186/s12913-019-4722-9

**Published:** 2019-12-04

**Authors:** Mesele Damte Argaw, Thandisizwe Redford Mavundla, Kassa Daka Gidebo

**Affiliations:** 1 USAID Transform: Primary Health Care, JSI Research & Training Institute, Inc., P.O. Box 1392, code 1110 Addis Ababa, Ethiopia; 2 Department of Health Studies, University of South Africa, Pretoria, South Africa; 3 School of Public Health, College of Health Sciences and Medicine, Wolaita Sodo University, Wolaita Sodo, Ethiopia

**Keywords:** Malaria, Clinical audit, Case management, Public–private partnership

## Abstract

**Background:**

Malaria is one of the leading public health problems in sub-Saharan Africa that contributes to significant patient morbidity and mortality. The aim of the study was to investigate adherence to malaria diagnosis and treatment guidelines by private health sector providers and compare their performance against the public private partnership (PPP) status.

**Methods:**

A facility-based retrospective clinical audit was conducted between October 2016 and January 2017 in 11 medium clinics in the West Gojjam zone of the Amhara Region, North-west Ethiopia. Data was extracted from patient medical records using pretested data abstraction forms. Descriptive statistics were employed to present the findings and adherence of health workers against the national and international standards were classified as *ideal*, *acceptable*, *minor error* and *major error* for both malaria diagnosis and treatment. A chi-square (X^2^) test was used to test for a statistically significant relationship after the data had been categorized using public private partnership status at *P* < 0.05.

**Results:**

One thousand six hundred fifty clinical files were audited. All malaria suspected patients were investigated either with microscopy or rapid diagnostics test (RDT) for parasitological confirmation. The proportion of malaria treated cases was 23.7% (391/1650). Of which 16.6% (274/1650) were uncomplicated, 3.69% (61 /1650) were severe and complicated and the rest 3.39% (56/1650) were clinical diagnosed malaria cases. And the malaria parasite positivity rate was 20.30% (335/1650). All malaria suspected patients were not investigated with ideal malaria diagnosis recommendations; only 19.4% (320/1650) were investigated with acceptable malaria diagnosis (public private partnership (PPP) 19.4%; 176/907; and non-public private partnership (NPPP) 19.38%; 144/743, X^2^ (1) = 0.0With regards to treatments of malaria cases, the majority 82.9% of *Plasmodium vivax* cases were managed with ideal recommended treatment (X^2^ (1) = 0.35, *P* = 0.55); among *Plasmodium falciparum*, mixed (*Plasmodium falciparum* and *Plasmodium vivax*).

**Conclusion:**

The clinical audit revealed that the majority of malaria patients had received minor error malaria diagnostic services. In addition, only one fifth of malaria patients had received ideal malaria treatment services. To understand the reasons for the low levels of malaria diagnosis and treatment adherence with national guidelines, a qualitative exploratory descriptive study is recommended.

## Background

In the last two decades, substantial progress has been made in fighting malaria [1]. According to the latest estimates of the World Health Organization (WHO), the incidence of malaria was reduced by 41% and the rate of malaria-associated deaths was reduced by 62% globally, between 2000 and 2015 [2]. However, at the beginning of 2016, malaria was still considered to be endemic in 91 countries and territories. Approximately 212 million cases of malaria and 429,000 deaths associated with malaria were reported in 2015 alone [2]. Malaria is prevalent in 75% of the 1.1 million square kilometre land mass of Ethiopia and affects over 60% of the Ethiopian population [3], which was estimated at 99 million in 2015 [4].

Globally, prompt and effective diagnosis and treatment of uncomplicated malaria cases has been implemented for several decades. This makes it possible for patients to be cured timely, preventing the development of severe malaria and subsequent death [5–8]. The current national malaria diagnosis guidelines recommend that every suspected case of malaria must be confirmed either by microscopy or by a rapid diagnostic test (RDT) before treatment is initiated [7–10]. Hence, anti-malarial drugs are prescribed only for confirmed cases. However, in areas where parasite-based diagnostic testing is not available, malaria treatment is initiated solely based on clinical suspicion. Therefore, parasitological confirmation is believed to improve the overall management of febrile illnesses [7].

**Fig. 1:**
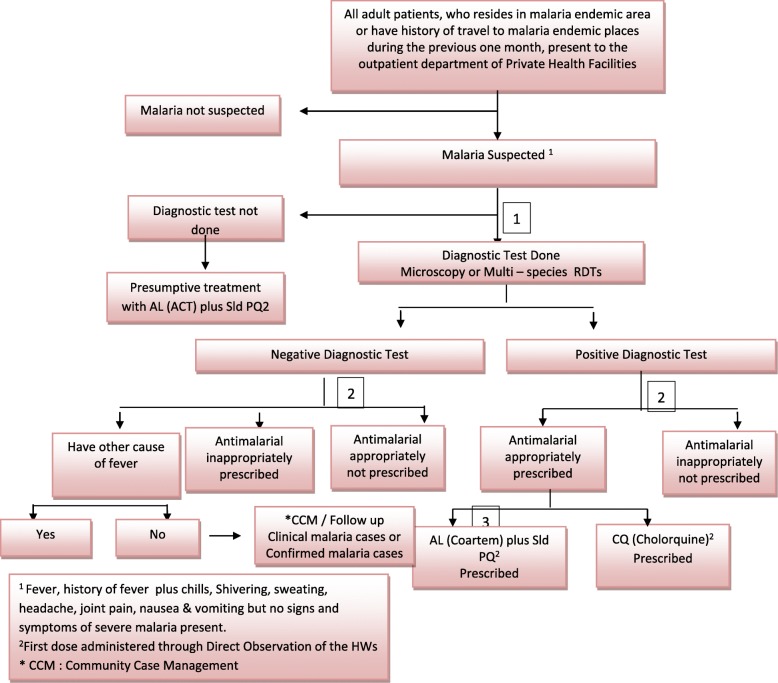
Algorism used to analyses adherence of health workers with the national & international standards, West Gojjam, Ethiopia, Oct 2016- Jan 2017. The figure clearly depicted the algorism of malaria diagnosis and treatment as stated in international national standards guidelines

The international and national malaria treatment protocols recommend treatment with species-specific anti-malarial drugs. The first-line recommendations for uncomplicated malaria include artemether-lumefantrine (AL), at a total dose of 5–24 mg/kg body weight (BW) of artemether and 29–144 mg/kg of lumefantrine plus 0.25 mg/kg BW single low-dose (SLD) primaquine, and chloroquine 25 mg/kg BW for the treatment of *Plasmodium falciparum* and *Plasmodium vivax* malaria, respectively. The second-line recommendation consist of quinine plus SLD primaquine for the treatment of uncomplicated *P. falciparum* or mixed or presumed malaria infection, and AL for the treatment of uncomplicated *P. vivax* malaria. No antibiotics are recommended for malaria case management in Ethiopia [7, 11]. Unlike the 2015 WHO treatment guidelines, Ethiopian malaria guidelines recommended radical cure of *P. vivax* malaria cases using primaquine 0.25 mg/kg BW/day for 14 days to be administered under health workers’ supervision only in malaria elimination target districts [11] (Fig. 1).

According to the third National Malaria Indicator Survey (EMIS 2015), close to one-fifth of malaria cases in Ethiopia were diagnosed and treated in formal private health facilities [12]. Among the ten zones of the Amhara Region, the West Gojjam Zone contributed the second largest number of malaria cases in the year 2013 [13]. Based on the routine health management information system (HMIS) report [14], there were about 14.01% (104,202/743,851) confirmed cases of malaria in 1 year, that is, July 2013 – June 2014 [15].

Effective malaria case management in both private and public health facilities will not only improve individual-centred care, but also prevents the transmission of diseases in the community and the emergence of ACT-resistant parasites [16, 17]. Accordingly, it is necessary to conduct a clinical audit of practices of actual case management of malaria in health facilities in comparison with the national and international standards.

In Ethiopia, public-private partnership approaches for malaria care services have been implemented since 2012 [18]. During the last 6 years, the PPM for malaria care service initiated with 39 private health facilities and the number of partner private health facilities reached 210 in 2016 [19]. The public health sector and public private partner facilities are part of quality assurance interventions which includes clinical audit, External Quality Assurance (EQA) scheme. Conducting operational research helps fill the information gap. This allows the public sector and the private health facilities to use the information to improve governance and stewardship to scale up the initiative so as to ensure access to quality and equity of malaria care.

The purpose of this clinical audit was to determine the adherence to standard malaria diagnosis and treatment guidelines by private health providers and compare results by partnership status among facilities located in the West Gojjam Zone of the Amhara regional state in the north-west of Ethiopia. Furthermore, the results of the study will be used to inform targeted interventions to improve the quality of outpatient malaria case management in the private health sector in Ethiopia.

## Methods

### Design of the study

A facility based retrospective study design was conducted through a clinical audit [20] of the diagnosis and treatment of uncomplicated malaria cases who were served in 11 medium clinics located in the West Gojjam Zone, Amhara, Ethiopia, between October 2016 and January 2017. This study period was selected for high malaria transmission season in the study areas.

### Setting of the study

The West Gojjam Zone is one of the ten administrative zones of the Amhara regional state. Based on the national census [21] (2007), the projected population of the West Gojjam Zone for 2017 was 2.2 million (www.CSA.gov.et). Four *woredas* (districts), namely, Finote Selam, Jabih Tehina, Bure and Wenberma were selected based on the reported high incidence of malaria cases. In West Gojjam zone health services are provided by one hospital, 90 health centres, 363 health posts and 76 private health facilities [15]. The clinical audit was carried out on patient medical records of 11 private for-profit medium clinics in the zone. All of the clinics sampled are established as private for-profit facilities, but 6 of the 11 facilities were working in public–private partnerships (PPPs) for malaria care services in terms of which partner facilities had access to anti-malarial supplies and received technical support from the public health sector [19].

### Population

The target population for this study was included of adults above 18 years of age who had been beneficiaries of malaria services in the targeted 11 medium clinics in the 3 months preceding the clinical audit. Only adults diagnosed and treated for uncomplicated malaria were included in this clinical audit. Since this clinical audit was conducted to explore the clinical practice and synthesize the information to develop an in-depth interview guide for qualitative research, patients under the age of 18 years, patients diagnosed with and treated for severe and complicated malaria, and patients with two or more diagnoses were excluded from the study.

### Sampling methods

The West Gojjam zone was selected through purposive sampling for its accessibility from the main road and convenience for the researchers. However, the *woredas*, the smallest administrative structures equivalent to districts, were selected based on the high burden of malaria. In 2012, the incidence of malaria in the selected four *woredas* ranged from 40 to over 100 per 1000 population [13]. All eleven medium clinics were enrolled in the study. Clinical records of patients with uncomplicated malaria were audited. The reviews were conducted in respect of the preceding three-month period. In each selected facility, all clinical files which fulfil the inclusion criteria were reviewed.

### Data collection tools and data quality

The data collection tools for the clinical audit were developed from the national and international malaria diagnosis and treatment guidelines [7, 8]. The tools developed for data collection were pretested using 30 clinical records of uncomplicated malaria in adult patients. Trained public health specialists, nurses and laboratory technologists were responsible for data extractions. In addition, one of the principal researchers supervised the day-to-day activities and ensured the consistency, completeness and quality of collected data.

### Extracted data

The data extracted from patient medical records and outpatient facility registers were used for this study. The data elements collected include: initial or follow-up visit; patient’s age, gender and weight; temperature; chief complaints; clinical features; methods of diagnosis; diagnostic tests; parasite load; and anti-malarial drugs prescribed and administered. The diagnosis and treatment criteria adopted for the clinical audit were proofed as “*Yes*”, “*No*”, or “*Not applicable*” as documented in the medical records. Methods of diagnosis were judged based on the findings “parasitologically confirmed using microscope”, “parasitologically confirmed using RDT” and “presumed malaria cases”. The laboratory investigation results, “*P. falciparum* malaria”, “*P. vivax* malaria” and “mixed infection (*P. falciparum* + *P. vivax* malaria)”, were extracted as documented in patient charts. Patient records with clinical and laboratory evidence of severe malaria, such as coma, severe malarial anaemia (haemoglobin or haematocrit), hypoglycaemia, circulatory collapse, renal failure (haemoglobin urea and creatinine), pulmonary oedema, spontaneous bleeding, repeated convulsions, acidosis, haemoglobinuria, impaired consciousness, jaundice, prostration, hyperpyrexia and hyperparasitaemia were excluded from the reviews for this study [7, 10].

For this study, medical records of 1650 adult outpatients who had received malaria services were audited in 11 medium private clinics. A clinical audit was systematically performed using selected information of patients who had received malaria services within the preceding 3 months (i.e. October 2016 – January 2017).

### Data analysis

Data entry and cleaning was conducted using Microsoft Excel 2010. For the statistical analysis, the cleaned data were exported to the Statistical Package for the Social Sciences (SPSS) (IBM-SPSS version 20). The descriptive results were presented using tables and graphs [22]. For categorical variables, a chi-square (X^2^) test was employed to test for a statistically significant relationship, which was claimed at *P* < 0.05.

### Ethical clearance

Ethical clearance was obtained from the Health Studies Higher Degrees Committee (HSHDC), College of Human Sciences (CHS), University of South Africa (UNISA). Prior to the commencement of data collection, the final version of the study protocol, together with the UNISA ethical clearance, was submitted to the Amhara Regional State Health Bureau, Research and Technology Transfer Core Process. Permission to conduct the research was obtained from the local Institution Review Board (IRB). In addition, a support letter was received from the West Gojjam zone Health Department. Consent to audit clinical records of randomly selected patients’ medical records was obtained from the heads or owners of all 11 medium clinics. To maintain the confidentiality of collected data, anonymity was maintained throughout the research process.

### Operational definitions

**Uncomplicated malaria** is defined as “a patient who presents with symptoms of malaria and a positive parasitological test (microscopy or RDT), but with no features of severe malaria” [7]. However, according to the Ethiopian national guidelines, presumed malaria cases can be identified in the absence of parasitological tests and parasitological negative result patients investigated for other causes of fever or managed through community case management (CCM) principles [11].

**Medium clinic** is the next level of health care to primary level in the health system providing ambulatory private health care that provides mainly curative, preventive and promotive services. According to the Ethiopian national minimum standards [23], a medium private clinic should be directed by a general practitioner with 3 years of relevant experience or by a public health officer or by a nursing practitioner with Bachelor of Science and who has 5 years of experience. To run a functional clinic, a minimum of six additional health personnel should be available in a single facility. The additional health personnel would commonly include 2 diploma nurses, 2 laboratory technicians, 1 radiographer and 1 midwife (optional).

#### Ideal malaria diagnosis

In accordance with the National Malaria Guidelines (2012), any adult patient suspect of malaria who is seen at a health facility should be tested for malaria parasites using thick & thin blood film, stained with 10% Giemsa for 15 min. The film would then be screened under an oil immersion microscope for the presence of *Plasmodium* spp., and parasite density would be determined as the number of parasites relative to the patient’s actual red cell count. If this is not available, an average red cell count of 5,000,000/μL of blood can be assumed in an *ideal malaria diagnosis* [7, 8, 11, 24–26].

### Acceptable malaria diagnoses

In the absence of a quality-assured malaria microscopy test, any adult malaria suspect patient would require testing with a malaria antigen test kit, which is a lateral flow immunochromatographic antigen detection test using finger-prick blood for rapid assay. Using this kit provides a rapid qualitative and differential test for detection of histidine rich protein-2 (HRP-2) or *Plasmodium* lactate dehydrogenase (pLDH) specific to *P. falciparum* and pan specific to other *Plasmodium* species (*P. vivax*, *Plasmodium malariae* or *Plasmodium ovale*). A unique positive HRP2 or PfpLDH line represents a *P. falciparum* infection whereas a unique panpLDH line indicates an infection with one or more of the non-falciparum species. The presence of both test lines indicates either an infection with *P. falciparum* or a mixed infection with *P. falciparum* and one or more of the non-*falciparum* species. In cases where the control line did not appear, the results were interpreted as invalid and the test repeated with a new device is an *acceptable malaria diagnosis* [7, 8, 11, 24–26].

### Minor error malaria diagnosis

An adult malaria suspect patient that is investigated using only Giemsa-stained thick film and has an estimated parasite density using semi quantitative + signs was categorized as *minor error malaria diagnosis* [7, 8, 11, 24–26].

#### Major error malaria diagnosis

An adult malaria suspect patient that is not identified and investigated for parasitological confirmation is considered as *major error malaria diagnosis* [7, 8, 11, 24–26].

#### Ideal treatment

If an adult has uncomplicated malaria then presumed malaria, *P. falciparum* and mixed (*P. falciparum* + *P. vivax*) infection is treated with artemether-lumefantrine (AL) at a total dose of 5–24 mg/kg body weight (BW) of artemether and 29–144 mg/kg of lumefantrine plus a single low dose (Sld) 0.25 mg/kg BW of primaquine. Patients with *P. vivax* infection are treated with chloroquine phosphate 25 mg/kg BW in three divided doses [11]. In Ethiopia, primaquine 0.25 mg/kg bw/day for a 14-day treatment for radical cure of *P. vivax* infection is recommended only in malaria elimination targeted districts [11].

### Acceptable treatment

Patients with presumed malaria that have *P. falciparum* or *P. vivax,* or a mixed infection, treated with second-line treatment recommended, AL or quinine for clinical malaria for *P. falciparum,* or mixed infection (*P. falciparum* + *P. vivax)*, and chloroquine plus primaquine for *P. vivax* infections, are considered as *acceptable treatment* [11].

### Minor error treatment

This term applies to a *P. falciparum* malaria patient, treated with either AL plus artemether injection, or to a *P. vivax* malaria patient, treated with chloroquine and artemether injection, or chloroquine and AL, or for both infections first-line drugs plus antibiotics prescription [11]. In addition, if the malaria patient risks to develop severe forms of malaria, or if a life-threatening situation is reduced, then the error which occurred only increases the cost for unnecessary drugs used at outpatient malaria management and was classified as *minor error* [27].

### Major error treatment

This term applies to malaria patients with *P. falciparum* or presumed malaria cases who are treated with only chloroquine and prescribed monotherapy (artemether) for *P. falciparum, P. vivax,* mixed or presumed cases [11]. In addition, if the risk for the malaria patient to develop severe forms of malaria or life-threatening situations is high, or treating malaria patients with monotherapy observed, then the error which occurred in outpatient malaria management was classified as *major error* [27].

## Results

### General characteristics of patients

In the 3 months that preceded this study, 1650 cases of suspected malaria were recorded in the outpatient facilities of the 11 medium clinics. Medical or clinical records were audited in respect of 330 adult patients who had been diagnosed with and treated for uncomplicated malaria. In addition, 61 clinical records (33 severe & complicated malaria; 28 malaria co-morbidity with other ill health conditions) were dropped from analysis. The mean age with standard deviation (± SD) of the assessed adult uncomplicated malaria patients was 29.9 (± 12.2) years. Their median age was 26 years and the age range was 63 (81–18) years. A total of 40.6% (*n* = 134) of the adult patients fell into the age category range 21 to 30 years. More than half (57.6%; *n* = 190) of the outpatient malaria service beneficiaries were males. More than half (55.2%) of the adult patients were from urban areas (Table 1).

**Table 1:** Demographic characteristics of uncomplicated malaria patient of the reviewed clinical records Oct- 2016- Jan 2017

Characteristics	Responses	Frequency	Percent
Age in categories	18–20 Years	89	27.0%
	21–30 Years	134	40.6%
	31–40 Years	48	14.5%
	41–50 Years	37	11.2%
	51 + Years	22	6.7%
	Total	330	100.0%
Mean (± SD): 29.9 (± 12.2)Years; Median: 26 Years; Range: 63 (81–18) Years			
Sex	Male	190	57.6%
	Female	140	42.4%
	Total	330	100.0%
Patient evaluated by partnership status (*n* = 11 facility)	PPP for malaria facilities	180	54.5%
	Non PPP for malaria facilities	150	45.5%
	Total	330	100.0%
Residence	Urban	182	55.2%
	Rural	148	44.8%
	Total	330	100.0%
District / *Woreda*	*Bure*	60	18.2%
	*Jabih Tehina*	60	18.2%
	*Wonberma*	90	27.3%
	*Finote Selam*	120	36.4%
	Total	330	100.0%

The table depicts the socio-demographic characteristics of selected patients

### Clinical history and physical examination

**Fig. 2:**
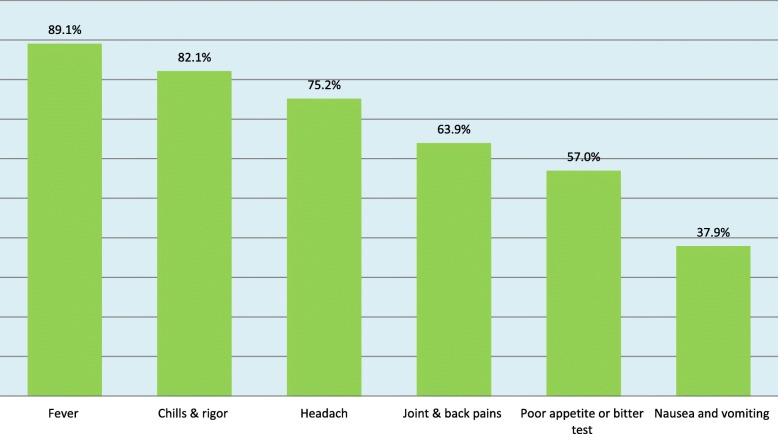
Bar chart showing proportion of clinical features, West Gojjam (*n* = 330), Oct 2016- Jan 2017. The figure depicts the frequency distribution of clinical features as documented in patients’ medical records

A review of the patient files revealed that chief complaints, detailed history of present illness and focused physical examinations had been well documented in 91.2, 88.1 and 95.2% of cases, respectively (Table 2). History of fever during the previous 2 days, feeling hot during physical examination or a temperature measurement > 37.5 °C was the most common (89.1%) clinical feature documented in the medical records of 294/330 selected uncomplicated malaria patients. Chills and rigor (82.1%) represented the second most common clinical feature and headache (75.1%) was the third most prevalent clinical feature (Fig. 2).

**Table 2:** Uncomplicated malaria patients’ clinical records and laboratory investigation, Oct 2016 – Jan 2017

Characteristics	Responses	Frequency	Percent
Chief compliant	Yes	301	91.2%
	No	29	8.7%
Comprehensive history	Yes	291	88.2%
	No	39	11.8%
Physical Examination	Yes	314	95.2%
	No	16	4.8%
Diagnosis methods (*n* = 330)	Blood Film (microscopy)	222	67.0%
	Rapid Diagnostic Tests (RDTs)	52	16.0%
	Presumed Diagnosis (Sign & Symptom)^a^	56	17.0%
Microscopy diagnosis results (*n* = 222)	*P. falciparum*	117	53.0%
	*P. vivax*	67	30.0%
	Mixed (*P. falciparum & P. vivax*)	38	17.0%
Rapid Diagnostic Tests results (*n* = 52)	*P. falciparum*	26	50.0%
	*P. vivax*	15	29.0%
	Mixed (*P. falciparum* or both *P. f* and *P. v*)	11	21.0%
Parasite load (*n* = 222)^b^	+	144	64.9%
	++	67	30.2%
	+++	9	4.0%
	++++	2	0.90%
Hemoglobin	Yes	254	76.9%
	No	76	23.1%
Urine analysis	Yes	182	55.2%
	No	148	44.8%

The table presents the frequency of records of clinical and laboratory investigations of adult uncomplicated malaria patients

^a^Fifty six patients were diagnosed for malaria based on evidences of signs & symptoms after having a negative reported result for parasitological tests

^b^Simple plus system method for estimating parasite load: + − 1- 10 parasites per 100 thick-film fields; ++: 11–100 parasites per 100 thick-film fields; +++: - 1- 10 parasites per thick-film field; ++++: more than 10 parasites per thick-film field [28]

### Diagnosis methods and results

This clinical audit also revealed that in 23.7% (391/1650) of suspected cases, malaria had been diagnosed. However, 3.69 (61/1650) medical records of severe and complicated malaria cases were excluded from further evaluation. The majority of malaria cases (83.0%; 274/330) had been confirmed by parasitological diagnosis, either by microscope or by malaria RDT. However, among parasitological negative patients, slightly less than one-fifth (17.0%;56/330) of adults with uncomplicated malaria had been diagnosed clinically after ruling out other causes of fever. In this study, all malaria species identification and parasite load estimation were made based on thick blood films.

### Laboratory investigations

It was found in the study that laboratory tests had been requested for all 1650 adult cases of suspected malaria. Blood tests for malaria using microscopy had been requested for the majority (80.6%; 1330/1650) of cases. On the other hand, one-fifth of cases (19.4%; 320/1650) had been investigated using malaria RDTs that is acceptable malaria diagnosis method, this performance does not show statistical difference by partnership status of targeted facility with 176/907 in PPP; and 144/743 in NPPP, X^2^ (1) = 0.0). The majority (80.6%; 1330/1650) of malaria suspected patients investigated for malaria diagnosis using only thick blood film was categorized as minor error diagnosis, this performance does not reveal statistical difference by partnership status with 170/1010 in PPP, and 80/320 in NPPP; X^2^ (1) = 1.92, *P* = 0.16). Moreover, no clinical records were found without investigation which fulfil the sign and symptoms to suspect malaria which were categorized as major error in malaria diagnosis.

The malaria parasite positivity rate was 20.3% (335/1650). Out of 222 microscopy-diagnosed malaria cases 117 (53.0%), 67(30%) and 38(17.0%) had been due to *P. falciparum*, *P. vivax*, and mixed *P. falciparum* plus *P. vivax* infections, respectively. Looking at the 52 malaria RDT diagnosed cases, 26 (50.0%), 15 (29.0%) and 11(21.0%) had been due to *P. falciparum*, *P. vivax,* and mixed *P. falciparum* plus *P. vivax* infections, respectively (Table 2)*.*

### Malaria case management

It was found in the study that the majority (82.9%; 68/82) of adult uncomplicated *Plasmodium vivax* malaria cases had received ideal treatment as per the standard recommendation using chloroquine phosphate 25 mg/kg BW in three divided doses (Table 3). This treatment regimen does not have significant differences (i.e. PPP: 82.2%; 37/45; and NPPP: 83.8%; 31/37) in adherence of national malaria standards by partnership status of enrolled private health facilities at X^2^ = 0.14; *P*-value = 0.70. However, not all *P. falciparum* or mixed (*P. falciparum* plus *P. vivax*) malaria cases had been treated with the ideal recommendation. Accordingly, the audit revealed that only one-fifth (20.6%; 68/330) of adult patients who had been treated for malaria had received the ideal recommended treatment.

**Table 3:** Anti-malarial prescription with ideal prescription or treatment classifications, West Gojjam, Oct 2016 – Jan 2017

Description	Ideal Treatment														
	AL+ sld PQ						Chloroquine 25 mg/kg bw						Over all		
	Illegible			Actual practices			Illegible			Actual practices			Illegible	Actual	Percentage
	Both PPM + NPPM	PPM	NPPM	Both PPM + NPPM	PPM	NPPM	Both PPM + NPPM	PPM	NPPM	Both PPM +NPPM	PPM	NPPM			
Microscopy (*n* = 252)															
*P. falciparum*	117	63	54	0	0	0	NA	NA	NA	NA	NA	NA	117	0	0.0
Mixed (Pf &Pv)	38	23	15	0	0	0	NA	NA	NA	NA	NA	NA	38	0	0.0
Presumed^b^	30	12	18	0	0	0	NA	NA	NA	NA	NA	NA	30	0	0.0
*P. vivax*	NA	NA	NA	NA	NA	NA	67	34	33	57	29	28	67	57	85.1
Sub total	185	98	87	0	0	0	67	34	33	57	29	28	252	57	22.6
Percentage				0.00	0.00	0.00				85.1	85.2	84.8		22.6	
RDTs (*n* = 78)															
*P. falciparum*	26	16	10	0	0	0	NA	NA	NA	NA	NA	NA	26	0	0.0
Mixed (Pf &Pv or Pf)	11	7	4	0	0	0	NA	NA	NA	NA	NA	NA	11	0	0.0
Presumed^b^	26	14	12	0	0	0	NA	NA	NA	NA	NA	NA	26	0	0.0
*P. vivax*	NA	NA	NA	NA	NA	NA	15	11	4	11	8	3	15	11	73.3
Sub total	63	37	26	0	0	0	15	11	4	11	8	3	78	11	14.1
Percentage				0.00	0.00	0.00				73.3	72.7	75.0			
Grand total	248	135	113	0	0	0	82	45	37	68	37	31	330	68	20.6
Percentage				0.00	0.00	0.00				82.9	82.2^a^	83.8^a^		20.1	

The table depicts the frequency of records of antimalarial drug prescription in 11 medium clinics in the West Gojjam Zone, Amhara. It also describes the ideal prescription or treatment practices by partnership status

^a^The X^2^ statistic is 0.14. The *p*-value is .70. This result is not significant at *p* < .05. Ideal treatment for Pf, Pf or Mixed & presumed malaria with AL plus Sld PQ was Zero; ideal treatment for Pv with CQ was 82.9% (68/82) and over all ideal treatment proportion was 20.6% (68/330).

^b^negative microscopy or RDT result but treated as presumed malaria cases

Approximately one-third (34.5%; 114/330) of patients had received second-line treatment using AL or quinine or chloroquine plus primaquine for *P. falciparum*, *P. vivax* or mixed infections. These prescription and practices were classified as acceptable treatment. This treatment regimen does not have significant differences (i.e. PPP: 32.8%; 58/180; and NPPP: 37.3%; 56/150) in adherence of national malaria standards by partnership status of enrolled private health facilities at X^2^ = 0.35; *P*-value = 0.55 (Table 4).

**Fig. 3:**
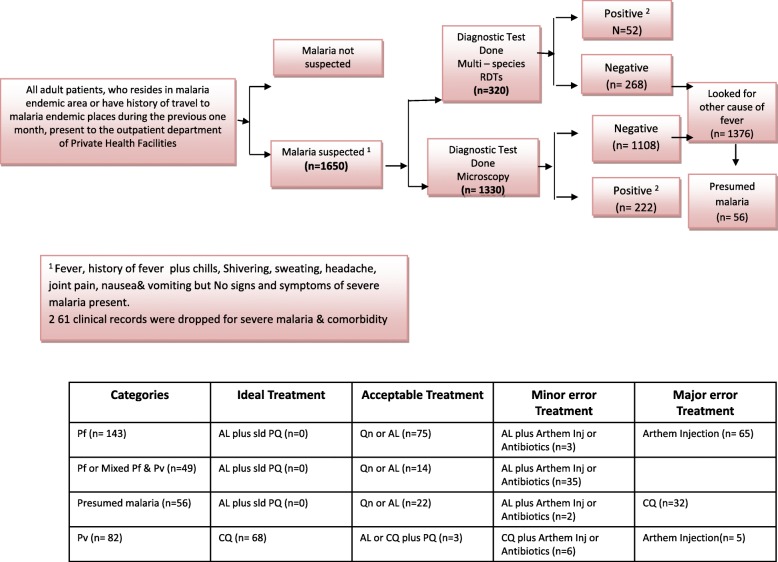
Clinical record audited against the standard malaria algorism, West Gojjam, Ethiopia, Oct 2016- Jan 2017. The figure summarize the findings of clinical record audited against the standard malaria diagnosis and treatment guidelines

This study showed that 13.9% (46/330) of adult patients had received AL plus an artemether injection, or chloroquine plus an artemether injection or AL, or chloroquine plus antibiotics (i.e. tetracycline, doxycycline, metronidazole, clarithromycin, cefotaxime) or AL plus chloroquine (Fig. 3). These treatment regimens fell in the “minor errors” category. This treatment regimen does not have significant differences (i.e. PPP: 13.3%; 24/180; and NPPP: 14.7%; 22/150) in adherence of national malaria standards by partnership status of enrolled private health facilities at X^2^ = 0.12; *P*-value =0.72.

Finally, slightly less than one-third of the reviewed medical records (30.9%; 102/330) indicated a diagnosis of *P. falciparum* or clinical malaria, where patients had been treated with chloroquine, or only using a mono-therapy prescription with an artemether injection. These practices were categorized under the “major errors” category. This treatment regimen does not have significant differences (i.e. PPP: 33.9%; 61/180; and NPPP: 27.3%; 41/150) in adherence of national malaria standards by partnership status of enrolled private health facilities at X^2^ = 1.16; *P*-value = 0.28 (Table 5).

**Table 4:** Anti-malarial prescription with acceptable and minor error treatment classifications, West Gojjam, Oct 2016 – Jan 2017

Description	Acceptable & Minor Error Treatments								
	Illegible			Acceptable treatment (AL or Qn Or CQ + 14 d PQ)			Minor Error (AL + Arth or Antib Or CQ + Arth or Antib		
				Actual practices			Actual practices		
	Both PPM + NPPM	PPM	NPPM	Both PPM + NPPM	PPM	NPPM	Both PPM + NPPM	PPM	NPPM
Microscopy (*n* = 252)									
*P. falciparum*	117	63	54	62	28	34	3	2	1
Mixed (Pf &Pv)	38	23	15	4	4	0	34	19	15
Presumed^a^	30	12	18	11	5	6	0	0	0
*P. vivax*	67	34	33	3	1	2	5	2	3
Sub total	252	132	120	80	38	42	42	23	19
Percentage				31.7	28.8	31.8	16.7	17.4	15.8
RDTs (*n* = 78)									
*P. falciparum*	26	16	10	13	9	4	0	0	0
Mixed (Pf &Pv or Pf)	11	7	4	10	6	4	1	1	0
Presumed^a^	26	14	12	11	5	6	2	0	2
*P. vivax*	15	11	4	0	0	0	1	0	1
Sub total	78	48	30	34	20	14	4	1	3
Percentage				43.6	41.6	46.6	5.1	2.1	10.0
Grand total	330	180	150	114	58	56	46	24	22
Percentage				34.5	32.8^b^	37.3^b^	13.9	13.3% ^c^	14.7%^c^

The table depicts the frequency of records of antimalarial drug prescription in 11 medium clinics in the West Gojjam Zone, Amhara. It also describes the magnitude of acceptable prescription or treatment practices by partnership status. In addition, it describes the frequency of minor errors treatment practices by partnership status

*AL* Artemether –Lumefantrine, *PQ* Premaquine, *CQ* Chloroquine, *Qn* Quinine, *sld* Single low dose, *Arthem* Arthemter injection, *Antibio* Antibiotics, ^a^: negative microscopy or RDT result but treated as presumed malaria cases; Acceptable Treatments: AL or Quinine for Pf malaria cases 75 patients; AL or Quinine for Mixed (Pf or Pf & Pv) malaria cases 14 patients; AL or CQ plus PQ for 14 days for 3 patients; Presumed malaria cases treated with Qn or AL for 14 patients. Minor Error treatment: AL plus Arthem Injection for 3 Pf malaria cases; CQ plus Arthem Injection for 6 Pv malaria case; AL plus Arthem plus Tetracycline or Metronidazole or Doxycycline 35 mixed (Pf & Pv) malaria cases; AL plus Arthem Injection for 2 Presumed malaria cases

^b^The X^2^ statistic is 0.35. The *p*-value is 0.55. This result is not significant at *p* < .05

^c^The X^2^ statistic is 0.12. The *p*-value is 0.72. This result is not significant at *p* < .05

**Table 5:** Anti-malarial prescription with major error classifications, West Gojjam, Oct 2016 – Jan 2017

Description	Major error Treatment					
	Major Error (CQ for Presumed Malaria Or Monotherapy with Arthem injection for Pf or Pv or mixed malaria cases)					
	Illegible			Actual practices		
	Both PPM + NPPM	PPM	NPPM	Both PPM + NPPM	PPM	NPPM
Microscopy (*n* = 252)						
*P. falciparum*	117	63	54	52	33^a^	19^a^
Mixed (Pf &Pv)	38	23	15	0	0	0
Presumed^d^	30	12	18	19	7^b^	12^b^
*P. vivax*	67	34	33	2	2^a^	0
Sub total	252	132	120	73	42	31
Percentage				28.9	31.8	25.8
RDTs (*n* = 78)						
*P. falciparum*	26	16	10	13	7^a^	6^a^
Mixed (Pf &Pv or Pf)	11	7	4	0	0	0
Presumed^d^	26	14	12	13	9^b^	4^b^
*P. vivax*	15	11	4	3	3^a^	0
Sub total	78	48	30	29	19	10
Percentage				37.2	39.6	33.3
Grand total	330	180	150	102	61	41
Percentage				30.9	33.9^c^	27.3^c^

The table depicts the frequency of records of antimalarial drug prescription in 11 medium clinics in the West Gojjam Zone, Amhara. The table presents the magnitude of major errors by partnership status

^a^70 Pf or Pv malaria patients were treated with only Arthem Injection;

^b^ 32 ^d^Presumed malaria patients were treated with CQ;

^c^The X^2^ statistic is 1.16. The *p*-value is 0.28. This result is not significant at *p* < .05; Major Error Treatment

## Discussion

Sarkar and Seshadri [20] describe clinical records review as a process that is aimed at obtaining retrospective data to answer clinical queries. They also state that this process has other known names such as ‘retrospective data analysis’, ‘clinical chart review’ and ‘chart review. Regular clinical audits provide a method for systematically reflecting on and reviewing practices. Changes can be implemented at an individual, team or service level [29]. This clinical audit revealed the current uncomplicated malaria case management practices in outpatient facilities of 11 medium private clinics in the 3 months that preceded the audit, that is, October 2016 –January 2017, in the West Gojjam Zone of the Amhara region, Northwest Ethiopia.

In this study, the general information of patients, evidence which includes initial or follow-up visit, age, blood pressure, pulse, dark colour of urine, generalized weakness (prostration) and jaundice, creatinine, and haemoglobin or haematocrit laboratory results from investigations were used to classify malaria cases as uncomplicated or severe and complicated. These criteria are in line with national and international classification either as uncomplicated or severe and complicated malaria cases [7, 8, 10].

In this study, history of fever 2 days prior to examination, feeling hot during examination or a measured temperature above 37.5 °C was recorded in the majority of cases of patients diagnosed with and treated for malaria. The most common clinical features like headache, chills, shivering, joint pain, backache and anaemia were checked, indicating that the high malaria investigation rate was optimal. In contrast with this finding, Meremikwu et al. [30] documented poor clinical records by private practitioners in Nigeria. However, this finding is consistent with evidence of improvement in the quality of public health services in the private sector through exercising public private partnership approaches as presented by Basu et al. [31], and Yimer and Yalew [18].

The malaria parasite positivity rate was 23.7% (391/1650). This finding is slightly lower than the four-year retrospective data analysis report by Argaw et al. [19] of a 24.5% malaria parasite positivity rate from 2959 facilities/month data; Legesse et al. [32] reported an average 33.3% malaria parasite positivity rate from 5 years health facility data; and Argaw [33] found a malaria parasite positivity rate of 37.6%. Despite diagnosing malaria using only thick film, the result of malaria EQA for 31 public private mix partnership engaged facilities score a concordance rate of 94% through regional research and a laboratory centres expert [34]. Therefore, the laboratory result is reliable. The difference might be explained by the differences in the study periods and study areas.

The audit documented that the majority of adults with malaria had been treated after parasitological confirmation through microscopy or RDTs, which was in line with the national and international recommendations. However, only one fifth of malaria patients had received ideal antimalarial prescriptions in line with the national and international recommendations [7, 8]. Adherence of ideal treatment does not show significant difference by public private partnership status. This finding might have occurred due to lack of uninterrupted supplies, and regular technical support by the regulating bodies. However, Basu et al. (2012) attribute the poor quality of work in the formal private sector in part to perceived incentives linked to unnecessary testing and treatment [31]. The result from this study also reinforces the finding that there is room for improvement regarding the efficient and effective use of antimalarial drugs and supplies in the private sector.

In all 11 private health facilities enrolled in the study, relative parasite load counts were used. This semi-quantitative method of quantifying using “+” signs is recommended by WHO (1991) for use only when it is not possible to undertake a parasite count per microlitre of blood [24]. This finding may be explained by lack of laboratory supply, that is, absolute methanol or ethanol to fix the thin blood film, as the parasite load result reported by all private health facilities employ were using only thick blood film smears. However, the WHO (2015), in the third edition of its guidelines for the treatment of malaria, discourages this method of parasite load estimation [7]. Moreover, thick blood film is recommended for screening suspected cases of malaria, while thin blood film is to be used to identify species of Plasmodium parasites, quantify proportion of parasitized red blood cells, perform platelets count and study blood cells morphology [25, 35].

In Ethiopia, the two most dominant malaria parasites are *P. falciparum* and *P. vivax* [3]. In this audit, slightly more than half (52%) of the parasitologically confirmed cases were caused by *P. falciparum*, close to one-fifth (17.8%) of the malaria cases were caused by mixed (*P. falciparum* plus *P. vivax*) infections, while the rest (30.0%) were caused by *P. vivax*. This finding is in line with proportions of reported malaria species. A nationwide facility retrospective report by Argaw et al. [19] indicates that 50.4, 45.6 and 4.1% of confirmed malaria cases were caused by *P. falciparum*, *P. vivax* and mixed infection, respectively. In addition, these figures were consistent with the report of the Ethiopia malaria indicator survey (EMIS), in which *P. falciparum* is reported to account for 77% (MIS 2011) [36] and 87.9% (EMIS 2015) [12] of the total reported malaria cases. The difference in figures might be explained by the reporting of *P. falciparum* cases only, or *P. falciparum* as mixed cases [3, 12, 37, 38].

In this study, no malaria patients were investigated with ideal malaria diagnosis methods recommended by WHO [25]. This could be occurred due to lack of laboratory supplies like absolute methanol or ethanol and the laboratory staff may not be motivated to engaged in demanding and time-consuming activities due to high workload. In addition, close to one fifth of uncomplicate malaria cases were investigated with acceptable malaria diagnosis method. Though this was helpful to improve species specific management, has some limitation on quantification of parasite density. The majority 80.6% of malaria patients were investigated using thick film which is reliable on screening and accepting negative results. Species identification and quantification of parasite load determines classification of patients as uncomplicated and severe life-threatening malaria cases [39]. Hence, it also reflects on evidence-based decision making on malaria patient management.

Most adults were treated for a parasitologically confirmed malaria diagnosis and only 82.9% *P. vivax* malaria patients received the ideal treatment. However, not all *P. falciparum*, mixed and presumed malaria patients were treated based on the recommended treatment regimen of the recently revised national malaria guidelines of Ethiopia, which is AL plus SLD primaquine. Furthermore, slightly less than one-fifth of presumed malaria patients were diagnosed and received treatment after negative laboratory test results had been documented. This finding is in line with Argaw’s [33] finding that the national survey revealed that health workers adhered to standard recommendations with respect to less than half of malaria patients they encountered. This could partly be due to insistence and pressure from patients to get anti-malaria drugs for febrile illnesses, providers’ clinical beliefs and capacity constraints of health providers to look for other causes of fever, limited patient diagnosis services and practices to identify other aetiologies such as viruses at medium clinics [28, 40, 41].

**Fig. 4:**
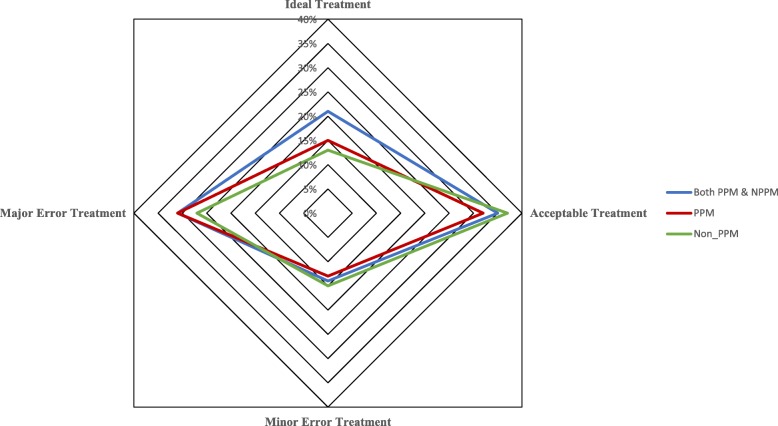
Radar chart showing the adherence to malaria treatment by partnership status, West Gojjam Zone, Amhara, Ethiopia, Oct 2016- Jan 2017. The chart depicts the comparative compliance with national and international guidelines by partnership status

Despite these deviations from national and international recommendations, more than one third of Ethiopian mothers preferred to visit private sector facilities for their perceived responsiveness than public sector ones. This finding was in line with the Awoke et al. [42] report on perceived better responsiveness of the private sector compared to the public sector in Ghana. Figure 4 depicts the comparative achievements by partnership status. More patients were given the ideal treatment, fewer second-line drugs were prescribed and there were fewer minor errors in PPP facilities than in non-PPP facilities. This finding was not consistent with the findings of [29, 43–45], who also documented evidence of improvement in quality achieved through working in various modalities of partnerships.

These major deviations from both national and international recommendations need intervention by both the private and the public sector, otherwise the poor quality of care documented in the private sector will contribute to drug resistance and loss of resources [17]. This finding is not in line with the standard recommendation on the management of uncomplicated malaria patients based on identified species.

## Limitations

This clinical audit was performed using retrospective data; as a result, there was a higher risk of getting incomplete and inconsistent data. In addition, it is imperative to consider the small sample size before interpreting and inferring the result of this study; the exclusion of patients age less than 18 years, diagnosed with severe and complicated malaria, and co-infected with other diseases may reduce the estimation on prevalence and species.

## Conclusions

The clinical audit revealed that the majority of malaria patients had received malaria diagnostic services with minor errors. In addition, only one fifth of malaria patients had received ‘ideal’ malaria treatment services. Therefore, efforts should be made to improve access to antimalarial supplies including absolute methanol, Primaquine and AL. In addition, enhancing the diagnosis and management capacity of healthcare providers though supervision and technical support are recommended. Finally, to understand the reasons for the low levels of malaria diagnosis and treatment adherence with national guidelines, a qualitative exploratory descriptive study is recommended.

## Data Availability

The datasets used and/or analysed during the current study are available from the corresponding author on reasonable request.

## Abbreviations

ACT: Artemisinin-based combination therapy
AL: Artemether-lumefantrine
CQ: Chloroquine
CSA: Central Statistics Agency
EFMOH: Ethiopian Federal Ministry of Health
EMIS: Ethiopia malaria indicator survey
EPHI: Ethiopian Public Health Institute
PPPs: Public–private partnerships
RDT: Rapid diagnostic test
RHB: Regional Health Bureau
UNISA: University of South Africa
WHO: World Health Organization
